# Neutrophil to Lymphocyte Ratio in Epilepsy: A Systematic Review

**DOI:** 10.1155/2022/4973996

**Published:** 2022-08-30

**Authors:** Samaneh Hosseini, Amir Mohammad Eghbalnejad Mofrad, Peyman Mokarian, Shima Nourigheimasi, Amir Azarhomayoun, Shokoufeh Khanzadeh, Saba Habibzadeh, Arshin Ghaedi

**Affiliations:** ^1^Neurosciences Research Center, Tabriz University of Medical Sciences, Tabriz, Iran; ^2^Ahvaz Jundishapur University of Medical Sciences, Iran; ^3^Fasa University of Medical Sciences, Fasa, Iran; ^4^School of Medicine, Arak University of Medical Sciences, Arak, Iran; ^5^Sina Trauma and Surgery Research Center, Tehran University of Medical Sciences, Tehran, Iran; ^6^Student Research Committee, Tabriz University of Medical Sciences, Tabriz, Iran; ^7^Clinical Research Development Unit of Children Hospital Tabriz University of Medical Science, Tabriz, Iran; ^8^Student Research Committee, School of Medicine, Shiraz University of Medical Sciences, Shiraz, Iran

## Abstract

This study was conducted to summarize the results of studies investigating the role of neutrophil to lymphocyte ratio (NLR) in epilepsy. The search was conducted on PubMed, Scopus, and Web of Science up to December 25, 2021. Finally, a total of seven studies were included in the review. The NLR in patients who were in the acute phase was higher than that of healthy. NLR in the patients who were in either acute or subacute phase was higher than in healthy controls. A significant difference in NLR levels between the acute and subacute phases was also noted. Epilepsy is one of the most important neurological diseases in the world, and millions of people around the world suffer from it, and a cheap and fast biomarker is needed for it. The interesting thing is that inflammation plays a role in epilepsy, and elevated NLR value can be a good biomarker of inflammation and, as a result, for epilepsy.

## 1. Introduction

Epilepsy is at the top list of common neurological disorders and is caused by an elevation in the stimulability of neurons in the brain [1, 2]. This brain disorder is marked by seizures and cognitive problems [3]. Epilepsy affects nearly 1% of the population around the world, which is about 70 million individuals, and 80% of those affected live in the middle- and low-income nations [4]. Inflammation has been shown to play a role in the initiation and development of epilepsy in animal models [5], and penetration of inflammatory cells in epileptic tissues has been discovered [6–9]. Leukocyte or lymphocyte and monocyte infiltration of the hippocampus and temporal cortex was discovered in an animal model with epilepsy and tissues taken from the temporal lobe of a person [6]. Epileptogenesis could be influenced by local and systemic inflammatory responses because, in cerebrospinal fluid and peripheral blood of epileptic patients, higher levels of chemokine and cytokines and elevated expressions of them can be observed [10, 11]. Some studies have shown the relationship between an elevated level of neutrophil to lymphocyte ratio (NLR) and several human disorders [12]. This ratio can be a crucial indicator for systemic inflammation, and it can be counted simply by taking a blood sample from patients [13–16]. Several studies indicate that increased NLR levels are associated with a worse prognosis or shorter survival in various malignancies, including brain glioma brain metastases, and are associated with disease development in inflammatory brain disease [14, 15, 17]. NLR has been introduced as a novel inflammatory predictor in various clinical conditions including cardiac arrhythmia [16], thyroid autoimmunity [18], type 2 DM [19], irritable bowel syndrome [20], COVID-19 infection [21], and other thyroid conditions [22]. Since epilepsy is also associated with inflammation [11], studying NLR in epilepsy is reasonable. If the relationship between increased NLR and epilepsy is proved, expensive equipment and materials for detecting and curing epilepsy will not be needed anymore. We performed a systematic review to consolidate all available information on the involvement of the NLR in epilepsy in order to see whether NLR is increased in patients with epilepsy or not and help clinicians better understand the epilepsy disease.

## 2. Methods

### 2.1. Search Strategy

We conducted our systematic review in compliance with the Preferred Reporting Items for Systematic Reviews and Meta-Analyses (PRISMA) guidelines. Two researchers independently searched three main databases (Web of Science, PubMed, and Scopus) for all relevant papers without regard to date or language constraints. When there were disagreements, a consensus was reached via group discussion. Using the keywords (“neutrophil-to-lymphocyte ratio” OR “neutrophil to lymphocyte ratio” OR “NLR”) AND (“epilep∗”), we searched the literature for studies on the diagnostic and prognostic usefulness of the NLR. On December 25, 2021, the search was finally updated. We also looked through the reference lists of the relevant research to see if there were any more papers.

### 2.2. Study Selection and Data Extraction

After removing duplicates, two researchers independently evaluated the titles, abstracts, and full texts of relevant articles. The following items were the criteria for inclusion:

1. Peer-reviewed original studies
2. Measuring the NLR by dividing the total neutrophil count by the total lymphocyte count
3. Reporting NLR value in peripheral blood
4. Assessment of the prognostic and diagnostic value of the NLR in epilepsy

The exclusion criteria were (a) in vitro and animal studies; (b) case reports, letters, editorials, and reviews; (c) studies that did not have enough data.

## 3. Results

### 3.1. Literature Search

**Figure 1:**
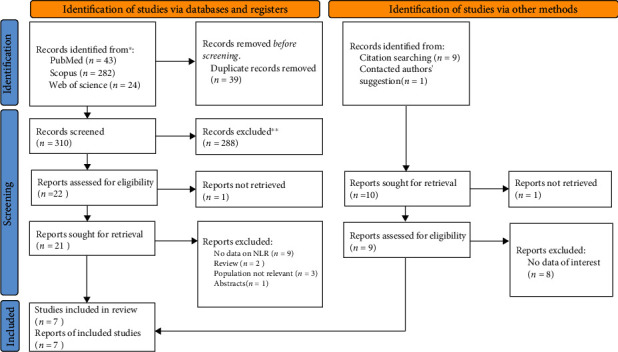
PRISMA 2020 flow diagram for new systematic reviews which includes searches of databases, registers, and other sources.

As illustrated in Figure 1, the database search and manual search of the article citation list yielded a total of 359 results. Finally, this systematic review included seven papers.  Table 1 shows the general characteristics of included studies. Of the included articles, five were retrospective, and two were prospective. We included 388 epileptic patients and 306 controls. The controls were people who admitted to the neurology clinics for reasons other than epilepsy.

### 3.2. NLR Role in Epilepsy

Güneş and Büyükgöl [23] conducted prospective research on the relationship between NLR and generalized epileptic seizure (in acute and subacute phase) in 2020. They studied 72 patients with generalized tonic-clonic epileptic seizures admitted to a neurology clinic in Aksaray, Turkey, and 72 healthy controls. There was no significant difference in age and sex between the two groups. NLR in patients who were in the acute phase was higher (4.46 ± 4.02) than that of healthy controls (1.81 ± 0.97, *p* < 0.001). NLR in the same patients who were in the subacute phase was higher (2.4 ± 1.34) than in healthy controls (*p* = 0.003). A significant difference in NLR levels between the acute and subacute phases was also noted (*p* < 0.001). This study also suggested that the probability of epileptic seizure increased 1.954 (1.335-2.859, *p* = 0.001) times for every unit rise in NLR during the acute phase and 1.731 (1.206-2.483) times for every unit rise in NLR during the subacute phase.

In parallel, Baran et al. [24] retrospectively observed 36 patients who had surgery for temporal lobe epilepsy (TLE) and 35 controls who admitted to their clinic for other reasons, and the MRI of control group was normal. Seven patients (19%) showed extensive hypometabolism on Positron emission tomography (PET), including temporal lobe and other areas of brain, and 18 patients (50%) had febrile seizures in the past. The goal of this study was to compare preoperative NLR levels in patients with temporal lobe epilepsy to controls and to evaluate the effect of febrile seizures and extending hypometabolism in brain on inflammatory biomarkers like NLR. There was no significant difference between patients and controls in gender (*p* = 0.74) and mean age (*p* = 0.51). NLR was higher in the patients (2.66 ± 3.70) compared to the controls (1.83 ± 0.49, *p* = 0.19). In the receiver-operating characteristics (ROC) curve analysis, the best cut-off value of NLR to distinguish between healthy controls and epileptic patients was 2.04 (test specificity = 74%, test sensitivity = 22%). And the area under the curve (AUC) for NLR was 0.43 (*p* = 0.33). In the TLE patients, febrile seizures can contribute in inflammation and may alter the NLR value, so this study divided the patients into patients with a history of febrile seizures (*n* = 18) and patients without febrile seizures (*n* = 18). Patients with a history of febrile seizures showed higher NLR value (3.08 ± 4.9) comparing to patients without febrile seizures (2.23 ± 1.7). However, this difference was not significant (*p* > 0.05). When comparing patients with and without a history of febrile seizures to controls, NLR revealed greater mean levels, although the differences were not significant (*p* > 0.05). This study evaluated the effects of extending hypometabolism in the brain on NLR, too. In seven patients, hypometabolism was observed in temporal lobe and other areas (“temporal-plus”), and in 29 patients, hypometabolism was observed only in the temporal lobe (“temporal lobe only”). NLR disclosed higher mean levels in the “temporal lobe only” group (2.95 ± 4) compared to “temporal-plus” group (1.47 ± 0.2), but this difference was not significant (*p* > 0.05). NLR was a little bit higher in the controls (1.83 ± 0.4) compared to temporal plus group, but it was insignificant (*p* > 0.05). NLR values were insignificantly higher in the temporal lobe only group when comparing to the controls (*p* > 0.05). Pearson correlation showed that there was a significant positive correlation between platelet lymphocyte ratios (PLR) and NLR (*p* = 0.00001). This study also showed that NLR correlated negatively with seizure frequency, history of febrile seizure, and extent of hypometabolism in the brain and positively with duration of seizure but none of them were significant (*p* > 0.05).

Similarly, Özdemir et al. [13] retrospectively recruited 58 CSE (convulsive status epilepticus) patients and 58 healthy controls to determine and compare the NLR values in acute and subacute phases. Of 58 CSE patients, 34 were GCSE (generalized convulsive status epilepticus), 13 were CPS-GSCE (complex partial secondary-generalized convulsive status epilepticus), and 11 were TSE (tonic status epilepticus). Within 6 hours and 72–96 hours following the commencement of the illness, NLR values were obtained from blood samples. Patients and healthy controls were sex- and age-matched (*p* > 0.05 for both). The average NLR value in the CSE patient group was 4.83 (±5.13) during the acute phase and 3.07 (±3.02) during the subacute period; however, it was 1.98 (±0.42) in the control group. There was a significant difference in NLR values between acute phase of CSE patients and healthy controls (*p* = 0.001) and also between subacute phase of CSE patients and healthy controls (*p* = 0.007). In GCSE patients, NLR value was 4.82 ± 0.86 in the acute phase and 3.50 ± 0.64 in the subacute phase; in the CPS-GCSE patients, NLR value was 5.14 ± 1.95 in the acute phase and 2.44 ± 0.36 in the subacute phase, and in the TSE patients, it was 4.49 ± 0.77 in the acute phase and 2.55 ± 0.44 in the subacute phase. Except for TSE patients, NLR levels were significantly higher amongst the subgroups of CSE patients compared to the control group in both the acute and subacute stages of CSE based on the ANOVA test (*p* < 0.05). The TSE patients and the control group had similar NLR levels (*p* > 0.05). Patients' antiepileptic medicines did not influence NLR levels in CSE patients (*p* > 0.05). During the acute phase of CSE, there was a moderate correlation between NLR and albumin levels based on the correlation analysis (*p* = 0.007, *r* = 0.248). During the subacute phase of CSE patients, a slight negative correlation between serum albumin levels and NLR was found (*p* = 0.004, *r* = 0.266).

Likewise, in the research published by Aslan and Cevik [25], one hundred thirteen patients from a neurology clinic in Medikalpark and 57 healthy individuals were retrospectively assessed. They conducted this study to compare crucial blood parameters such as NLR between epileptic patients and healthy controls. No statistically significant difference was observed in terms of gender and age (*p* = 0.745 for age and *p* = 0.359 for gender). NLR values were statistically significantly higher in the patient group (3.26, 0.1-14.5) than the control group (1.77, 0.5-23.0, *p* = 0.16). The likelihood of an epileptic seizure increased 1.239 (1.007-1.525, *p* = 0.043) times for every unit increase in NLR, according to this study.

These four studies suggested that NLR value was higher in epilepsy patients in compared to the healthy controls but the next three studies found different results.

In contrast to the previous studies, Ozdemir et al. [26] prospectively studied a total of 21 epileptic patients who were going to have an epilepsy surgery and 21 healthy controls in 2019. They conducted this study to determine NLR values in patients undergoing surgery. Patients mainly had focal onset seizures (14 of 21), and all of the patients were receiving AEDs. Most of the patients underwent right-sided TLE (temporal lobe epilepsy) surgery. There was no significant difference in age and sex between the two groups (*p* = 0.88 for age and *p* = 0.75 for gender). Blood samples were taken from the patients twice before surgery and again one week later, but blood samples were obtained from the controls once. NLR value was 1.93 ± 0.83 in the before surgery samples of patients and 1.96 ± 0.63 in the controls. There was no significant difference between these two groups (*p* = 0.89). NLR was 5.88 ± 3.72 in the after surgery samples of the patients, and there was a significant difference between after surgery samples and healthy controls (*p* < 0.01). Also, there was a significant difference in the NLR value between before surgery and after surgery samples of the patients (*p* < 0.01). So, surgery resulted in large increases in NLR, and NLR was significantly higher after surgery. This study also revealed a significant positive correlation between preoperative PLR (platelet lymphocyte ratio) and preoperative NLR (*p* = 0.00001, *r* = 0.72). A nonsignificant positive correlation between seizure duration, seizure frequency, and NLR was also observed (*p* > 0.05).

Eroglu et al. [27] published retrospective research including 52 patients with epilepsy and 49 healthy control subjects who admitted to their clinic for routine tests in 2017. They tried to compare NLR values between epilepsy patients during seizures and seizure-free period and healthy controls. In terms of sex and age, there was no significant difference between epilepsy patients and healthy controls (both *p* > 0.05). The NLR value was 1.84 (0.41-12.33) in the epileptic group during seizure and 1.89 (0.75-4.59) in the control group. Between epilepsy patients during the seizure period and controls, NLR was not significantly different (*p* = 0.959). During the seizure-free period in the epilepsy patients, NLR was 1.69 (0.71-6.13) and in the control group was 1.87 (0.75-4.59). No significant differences were observed in this phase (*p* = 0.28). NLR value was not significantly different between seizure-free phase and during seizure phase in epilepsy patients (*p* = 0.145).

Morkavuk et al. [4] conducted a study on 50 patients, 14 with psychogenic nonepileptic seizures (PNES) and 36 with epilepsy. Twenty-one epilepsy patients experienced generalized seizures, whereas 15 had seizures with a focal onset. By measuring the NLR value in the pre- and postseizure phase in epilepsy and PNES, they tried to identify seizures from pseudoseizures in this study. In generalized onset seizures, the male gender was shown to be dominating, and this was statistically significant (*p* = 0.045). There was no significant difference between the groups when the mean age was evaluated. In preseizure phase, NLR value was 1.81 (0.88-3.71) in generalized onset epileptic seizure group, 2.16 (0.83-3.67) in focal onset epileptic seizure group, and 1.51 (0.84-3.64) in PNES group. There was no significant difference in NLR value between preseizure groups (*p* = 0.364). In the postseizure phase, NLR value was 1.62 (0.63-10.71) in generalized onset epileptic group, 1.5(0.72-3.24) in focal onset epileptic group, and 1.44 (0.93-2.75) in PNES group. There was no significant difference in NLR value between postseizure groups (*p* = 0.761). In either group, NLR found no statistically significant change between pre- and postseizure levels (*p* = 0.794 for pre- and postseizure generalized onset epileptic seizure group, *p* = 0.061 for pre- and postseizure focal onset epileptic seizure group, and *p* = 0.397 for pre- and postseizure PNES group).

## 4. Discussion

The NLR in patients who were in the acute phase was higher than that of healthy. NLR in the patients who were in either acute or subacute phase was higher than that in healthy controls. A significant difference in NLR levels between the acute and subacute phases was also noted. As discussed earlier, epilepsy is one of the most important neurological diseases in the world, and millions of people around the world suffer from it, and a cheap and fast biomarker is needed for it [28]. The interesting thing is that inflammation plays a role in epilepsy, and elevated NLR value can be a good biomarker for inflammation and, as a result, for epilepsy [29, 30]. In a published study in 2020, it was discovered that every one-unit increase in NLR increased the incidence of epileptic seizures by 1.23 [25]. Güneş and Büyükgöl found that the probability of epileptic seizures increased 1.95 times for every unit rise in NLR [23]. Some studies claimed that NLR correlates with the intensity of systemic inflammatory disorders like multiple sclerosis and Behçet's syndrome [31, 32]. It is also crucial to understand the role of neutrophils and lymphocytes in epilepsy to acknowledge the relationship of this ratio (NLR) and epilepsy disease. In fact, NLR is determined as a simple ratio between proinflammatory cells, neutrophils, regulatory immune cells, and lymphocytes. As a result, a higher NLR indicates a higher degree of inflammation, contributing to epilepsy development. Previous research on the involvement of neutrophils in epilepsy has been extensive. However, the impact of lymphocytes is not fully understood, and further research is needed in this area. Neutrophils are the most abundant leukocytes involved in innate immunity, and the level of neutrophil cells increases in systemic inflammation. A study published in 2021 observed that the number of neutrophils was considerably higher in the postseizure phase than the preseizure phase in the group with generalized epilepsy [4]. Özdemir et al. found an association between the status epilepticus and neutrophil-mediated inflammation [13]. Moreover, in a study published by Güneş and Büyükgöl, it was detected that the number of neutrophil cells and NLR values were significantly higher in the acute period in comparison to the subacute period in patients with generalized epilepsy [23]. These data validate the concept that epileptic seizures are linked to neutrophil-mediated systemic inflammation. The results of a study published in 2021 suggest that TNF*α* and neutrophils regulate neuronal hyperexcitability, which is associated with a variety of etiologies [33]. This is important since Morkavuk et al. claimed that increased excitability of neuronal cells might cause epilepsy disease [4]. So, another connection between elevated neutrophil numbers and epilepsy disease can be seen. Apart from releasing neuroexcitatory cytokines, neutrophils may indirectly affect neuronal hyperexcitability by releasing chemokines that attract other inflammatory cells. Both CCL3 and CCL2, which were shown to be substantially elevated in epileptic brain tissue, are generated by neutrophils and help bone marrow-derived cells and monocytes to recruit [33]. Epilepsy often goes along with an increase in leukocytes, like neutrophils, in the hippocampus. The more these cells infiltrate into the hippocampus, the more neurons degenerate. The reason behind the infiltration and leakage of neutrophils and leukocytes into the hippocampus is blood-brain-barrier dysfunction and interactions between leukocytes and endothelium. So, one way to prevent seizures is to inhibit vascular-leukocyte interactions based on experimental studies. Infiltration of neutrophils and leukocytes can cause a rise in inflammatory mediators, including COX-2, complement, and tumor necrosis factors, which can explain why seizures happen in epilepsy disease [11]. The other part of the NLR ratio is lymphocytes. In a study published in 2020, it was observed that lymphocytes were significantly lower in epilepsy patients compared to controls, and also, lymphocytes were substantially lower in the acute phase in comparison to the subacute phase in patients [23]. Furthermore, Özdemir et al. found that lymphocytes are significantly lower in both the acute and subacute phases of epilepsy patients compared to controls [13]. Another study confirms that lymphocytes are significantly lower in patients with temporal lobe epilepsy compared to the controls [24]. So, we found out that neutrophil numbers increase and lymphocyte numbers decrease in epilepsy patients. This explains why the NLR ratio increases in these patients, and this is why NLR can be a potential biomarker for epilepsy as it is in other diseases like cancer [34].

## 5. Limitations

This systematic review has some limitations which are crucial to address. First and foremost, the included studies had a small sample size which led to some discrepancies. Also, our results may not be powerful enough to make a concrete conclusion regarding such values. Further studies with a larger sample size will be needed to solidify the relationship between NLR and epilepsy disease. Second, most of the included studies were retrospective. The results of retrospective studies may not be as reliable as prospective studies because retrospective studies are based on medical records in the past, and we are not aware whether or not these records are accurate. We do not know how these data are collected that can be questioned. Third, all of the included studies were conducted in Turkey. NLR values may vary according to race and such variations. So, further studies in different countries should be performed to prove or disprove the effect of race on NLR value. Among included studies, more than one type of epilepsy was studied, and some studies included specific or rare types of epilepsy, which can explain why some studies demonstrated that there is not a significant difference in NLR values between epilepsy patients and healthy subjects.

## 6. Conclusion

Our study supports the idea that there is a relationship between NLR values and epilepsy illness, and this ratio increases in epilepsy patients. Our results showed that NLR might be a promising biomarker that can be obtained easily with a simple blood test. Such new biomarkers and therapeutic interventions will help us better prevent and treat epilepsy, lowering long-term morbidity and death.

## Figures and Tables

**Table 1:** General characteristics of included studies.

First author	Year	Region	Design	Sample size	Mean age	Gender (percentage of males)	Main result
Aslan [25]	2020	Turkey	Retrospective	Total: 170 Patients: 113 Controls: 57	Patients: 51.61 ± 21.3 Controls: 52.86 ± 21.10	Patients: 55 (48.7%) Controls: 32 (56.1%)	There was significantly higher NLR in epileptic patients compared to the healthy controls.
Baran [24]	2020	Turkey	Retrospective	Total: 71 Patients: 36 Controls: 35	Patients: 33.6 ± 9.6 Controls: 32 ± 10	Patients: 20 (55.5%) Controls: 19 (54.28%)	NLR was not significantly different between patients and healthy controls.
Güneş [23]	2020	Turkey	Retrospective	Total: 144 Patients: 72 Controls: 72	Patients: 47.4 ± 20.5 Controls: 49.2 ± 15.6	Patients: 35 (48.6%) Controls: 36 (50%)	The probability of epileptic seizure increased 1.954 times for every unit rise in NLR during the acute phase and 1.731 times for every unit rise in NLR during the subacute phase.
Özdemir [13]	2016	Turkey	Retrospective	Total: 116 Patients: 58 Controls: 58	Patients: 34.6 ± 2.5 Controls: 37.4 ± 1.8	Patients: 26 (44.8%) Controls: 31 (53.4%)	NLR was not significantly different between patients with convulsive status epilepticus and healthy controls.
Morkavuk [4]	2020	Turkey	Prospective	Total: 50 Epilepsy: 36 PNES: 14	Epilepsy patients: 26.5 (18.5-48.3) PNES group: 33.5 (18–56)	Epilepsy patients: 23 (64%) PNES group: 4 (29%)	NLR is not a good biomarker for differentiating between patients with epileptic seizures and those with pseudoseizures.
Ozdemir [26]	2019	Turkey	Prospective	Total: 42 Patients: 21 Controls: 21	Patients: 30.14 ± 7.5 Controls: 29.76 ± 9.2	Patients: 9 (42%) Controls: 13 (61%)	Surgery for temporal lobe epilepsy resulted in large increases in NLR, and NLR was significantly higher after surgery compared to before surgery samples of patients.
Eroglu [27]	2017	Turkey	Retrospective	Total: 101 Patients: 52 Controls: 49	Patients: 31.57 ± 8.82 Controls: 33.54 ± 7.07	Patients: 20 (38.5%) Controls: 19 (38.8%)	NLR was not significantly different between patients and healthy controls.

PNES: psychogenic nonepileptic seizures; NLR: neutrophil to lymphocyte ratio.

## Data Availability

All data generated or analyzed during this study are included in this published article.
